# Microtissues in Cardiovascular Medicine: Regenerative Potential Based on a 3D Microenvironment

**DOI:** 10.1155/2016/9098523

**Published:** 2016-03-17

**Authors:** Julia Günter, Petra Wolint, Annina Bopp, Julia Steiger, Elena Cambria, Simon P. Hoerstrup, Maximilian Y. Emmert

**Affiliations:** ^1^Institute for Regenerative Medicine, University of Zurich, 8044 Zurich, Switzerland; ^2^Division of Surgical Research, University Hospital of Zurich, 8091 Zurich, Switzerland; ^3^Heart Center Zurich, University Hospital of Zurich, Zurich, Switzerland; ^4^Wyss Translational Center Zurich, Zurich, Switzerland

## Abstract

More people die annually from cardiovascular diseases than from any other cause. In particular, patients who suffer from myocardial infarction may be affected by ongoing adverse remodeling processes of the heart that may ultimately lead to heart failure. The introduction of stem and progenitor cell-based applications has raised substantial hope for reversing these processes and inducing cardiac regeneration. However, current stem cell therapies using single-cell suspensions have failed to demonstrate long-lasting efficacy due to the overall low retention rate after cell delivery to the myocardium. To overcome this obstacle, the concept of 3D cell culture techniques has been proposed to enhance therapeutic efficacy and cell engraftment based on the simulation of an in vivo-like microenvironment. Of great interest is the use of so-called microtissues or spheroids, which have evolved from their traditional role as in vitro models to their novel role as therapeutic agents. This review will provide an overview of the therapeutic potential of microtissues by addressing primarily cardiovascular regeneration. It will accentuate their advantages compared to other regenerative approaches and summarize the methods for generating clinically applicable microtissues. In addition, this review will illustrate the unique properties of the microenvironment within microtissues that makes them a promising next-generation therapeutic approach.

## 1. Introduction

Stimulated by the aging population, intense effort has been invested in the development of new strategies to address medical challenges [1]. Although the human body has lifelong regenerative potential by recruiting progenitor cells to replace lost cells through proliferation and differentiation, the endogenous regenerative capacity is limited and often insufficient after extensive tissue injury. Therefore, conventional treatments and interventions are primarily symptomatic and are usually not capable of curing the disease [2]. To achieve full recovery, it is critical to address the problem at its source by removing the cause and not merely postponing the consequences of tissue degeneration by applying symptomatic therapies. In this setting, regenerative medicine using stem and progenitor cells has emerged as a new and promising field [3]. The main objective is to replace damaged cells and, therefore, to restore the physiological structure and functionality of the diseased organs [4]. With stem cells as key players, the domain of regenerative medicine is continuously expanding from single-cell injections to the engineering of larger tissue implants that also include extracellular matrix (ECM) embedding of an entire arrangement of cells [5].

Cardiovascular diseases have been of particular interest for innovative (translational) therapy options because they could benefit significantly from a regenerative approach. The heart is known to have a limited capacity for self-regeneration [6, 7]. Current therapies after myocardial infarction (MI) mainly involve pharmaceutical approaches and surgical or percutaneous revascularization. Unfortunately, ischemic heart disease is progressive, and the loss of cardiomyocytes provokes further remodeling processes that negatively affect the course of the disease [8, 9]. Therefore, contemporary options are only palliative and delay the fatality of the pathologies, which could ultimately only be averted with heart transplantation. However, an organ shortage and the necessity for lifelong immunosuppressive therapy significantly limit the application of this therapy option to a subset of patients [10, 11].

Over the past decade, several cell types have been examined for their capacity to repair and regenerate the heart. Bone marrow, and particularly its collection of subpopulations, including mesenchymal stem cells (MSCs) and endothelial progenitor cells (EPCs), has been evaluated intensively. In addition, other attractive sources, such as adipose tissue or the umbilical cord, could harbor a reservoir of suitable stem cells for cardiac repair. Furthermore, the detection of inherent progenitor cells in the human heart pioneered the development of next-generation approaches involving cardiac-derived stem cells from the heart itself as well as stem cells guided toward differentiation along the cardiac lineage ex vivo [5, 8, 12–14]. Many cell types have demonstrated promising results in preclinical trials, and clinical pilot studies have proven that these methods are feasible and safe, thereby raising hope for curing the human heart. However, in regard to efficiency, the outcomes were disappointing, and the minimal benefit showed that the strategies needed to be revised [5, 10, 15]. Several critical but controversial factors are responsible for the lack of success. Among the most fundamental points of debate are the optimal cell type, the delivery method, and the timing. A main focus must also be on the cell format [3, 10, 11]. Cells can be injected intracoronary or directly into the myocardium. Regardless of the route of delivery, one essential obstacle appears to be engraftment [5, 15, 16]. To exploit their full potential, cells need to be retained at the site of injury, and their long-term survival is a prerequisite to enable them to fully operate [10]. The acute retention rates that were observed did not exceed 10% [17]. Therefore, the cell format appears to play a pivotal role in impeding the high washout phenomenon of the current standard approaches [18]. Consequently, the strategy for the perfect implant has increasingly shifted to graft cells in a three-dimensional (3D) design, which more closely mimics living tissue. Compared to single-cell suspensions, stem cells can also be transplanted as complex tissue constructs to improve retention [5, 19]. However, it remains very challenging to design larger 3D cell constructs and, importantly, to maintain their viability. In addition, instead of simple, catheter-based injection, they often require more invasive surgery to be delivered [3]. A potential solution may be small, scaffold-free, 3D aggregates called microtissues. Microtissues include between 500 and 10,000 cells and were initially used as a model to elucidate tumor biology [5, 20]. However, as an interesting concept for 3D cell culture, their application has been extended to the area of regenerative medicine during the last several years [21]. In the setting of cardiovascular applications, microtissues may provide an optimal hybrid format to implant stem cells into the injured heart. They can be transplanted via transcatheter delivery because of their scaffold-free concept [3]. Compared to dissociated cells, their superior size and higher organized 3D state, along with their inherent ECM, promote enhanced integration and retention in the target tissue [17, 22]. In addition, they exhibit versatile advantages over their single-cell counterparts that are attributed to their 3D microenvironment, which also better reflects the native situation in vivo [21]. Therefore, microtissues have been repeatedly suggested to be the missing link for enhancing the therapeutic potential of stem cell therapy and bringing regenerative medicine a step closer to clinical application in cardiovascular medicine [10].

This review article summarizes the different aspects of microtissues in the context of cardiovascular regeneration. In particular, it highlights the technical and functional advantages of a scaffold-free 3D cell culture compared to other cell-based regenerative approaches, including scaffold-based and single-cell approaches. It emphasizes production strategies for microtissues for therapeutic purposes and notes remaining challenges to providing a product that is immediately available and accessible to a broad subset of patients. Furthermore, this paper introduces the remarkable cues of the in vivo-like microenvironment established in microtissues, relates these cues to resulting therapeutic advantages for cardiac regeneration, and refers to recent preclinical studies that have addressed cardiovascular therapy with microtissues. Finally, the potential of microtissues as building blocks for the generation of larger tissues is illustrated. As shown in this review, merging state-of-the-art techniques into an innovative, hybrid concept might result in a promising approach for using microtissues for advanced and targeted cardiac regeneration in the clinical arena.

## 2. Scaffold-Free versus Scaffold-Based 3D Tissue Culture

To understand the interest surrounding microtissues in the regenerative field, it is important to compare them with other tissue-engineering strategies, particularly the ones that include the use of a scaffold.

### 2.1. 3D Culture

Cells in physiological tissue are part of a versatile and dynamic network that can never be mimicked by a monolayer cell culture [23]. To cultivate cells in a 3D environment, there are different approaches available. Tissue culture can be based on a scaffold that serves as a platform for cell attachment and as a carrier for cell delivery [24], or the cells themselves can serve as the platform to guide cell aggregation to microtissues [25]. There are several advantages to providing cells in a 3D format:

- Microenvironment preserves physiological cell phenotype and gene expression profile.
- Cell-cell interaction plays a crucial role in cellular homeostasis.
- ECM enhances integration and retention in target tissue.
- Hypoxic environment induces growth factor expression.

This format better mimics the physiological situation because cells in living tissues are normally anchored to other cells or to the ECM [25]. These adhesions not only provide mechanical anchor points but also influence cell behavior through signaling properties of both components [26–28]. The comparison of cell cultures in 2D and 3D highlights the improved cell functionality [29, 30] and regenerative capacity [29, 31] created by 3D cultivation. In addition to the increased physiological behavior, functionality, and vitality of cells in 3D tissues, the elevated secretion of ECM can enhance cell viability and provide therapeutic advantages [29, 32].

### 2.2. Scaffold-Based Approaches

The two main concepts of tissue engineering are the development of tissues with or without scaffolds. Both have advantages and disadvantages, and the strategy to engineer the ideal graft depends primarily on the needs and features of the desired tissue (Table 1). Scaffolds can serve as an ECM mimetic, which can subsequently home the cells. Because the ECM is a very complex and versatile structure, it is difficult to mimic it optimally. Frequently, biodegradable polymers have been used as scaffolds and can be of either natural or synthetic origin [33]. Both scaffold sources are made of external materials, which may cause rejection and foreign body reactions as well as overgrowth by fibroblasts [3]. While synthetic scaffolds may have a lower risk of immunogenicity, there are concerns about the biocompatibility of their degradation products [33]. Scaffolds can be modified and activated in a nearly unlimited way by modulating the surface chemically [34] or physically [35] or by coupling biologically active molecules [36–38]. Possible benefits of scaffold-based approaches are their strength and mechanical support, their available capacity to adapt in size to large areas of tissue damage, their guidance or preorganization of cell growth and tissue shape, and the potential addition of bioactive proteins and molecules [33]. For complex tissues, such as cardiac tissues, many aspects have to be considered for the scaffold design because the native myocardial tissue permanently switches between contraction and relaxation, causing a high level of oxygen consumption [33]. The main drawback of scaffold-based engineered tissues is the delivery to the target organ or structure. Larger tissues may not be applicable for a minimally invasive technique and may require open surgical approaches, such as open-heart surgery, when addressing cardiac tissues [3].

### 2.3. Scaffold-Free Approaches

In contrast to scaffold-based strategies, tissue engineering without scaffolds is based on the self-assembly of cells. Two established forms of scaffold-free tissues are cell sheets and microtissues. Cell sheets can be produced via 2D cultivation of cells on special surfaces that allow the detachment of the whole monolayer [39]. Disadvantages of these sheets are the difficulty in handling them due to their thinness and the lack of catheter-based applicability [33]. Microtissues can be produced by various methods that promote the self-aggregation of cells to spheroids [26]. Their handling appears to be easier, and they are injectable via a transcatheter delivery technique [10], which is a significant advantage for clinical applications. Because of their more native-like structure, they are capable of an elevated secretion of ECM and paracrine factors [29]. Moreover, compared to scaffold-based tissue-engineering approaches, the use of microtissues circumvents obstacles, including intense material investigation for the optimal scaffold, the biocompatibility of the graft and its degradation products, and scaffold degradation capacity [33].

For these reasons, microtissues have emerged as an attractive alternative to scaffold-based strategies in the field of tissue regeneration.

## 3. Production of Therapeutic Microtissues

While 3D, scaffold-free microtissues have several advantages for regenerative therapies, their production has to be compliant with clinical requirements, including simplicity, reduced cost, product quality, standardization, and the possibility of upscaling. This chapter will analyze the different existing methods coming from the fields of both basic and clinical research in terms of these requirements. Insights into automation and biobanking will then be provided.

### 3.1. Methods for Microtissue Generation

Many approaches are available for the production of microtissues. They have the common principle of culturing cells in an environment where cell-cell interactions prevail over cell-surface interactions [40]. However, they all have advantages and disadvantages regarding clinical requirements.

First, a simple and clinically compliant method of forming microtissues without any external constraints occurs in the cardiovascular field with cardiospheres. Cardiac biopsies are enzymatically digested, yielding explants that are cultured under specific conditions. Cells migrating from the explants are harvested and spontaneously form niche-like spheroids after seeding [41]. Remarkably, cardiosphere-derived cells (CDCs) can be plated to form secondary cardiospheres, and they possess significant cardiogenic potential [42]. CDCs are currently considered to be among the most promising cell types for cardiac regeneration, as shown by the successful results of phase I clinical trial CADUCEUS [43]. We suspect that the combination of CDCs/cardiospheres with microtissues composed of other cell types, such as MSCs, might even enhance this effect. However, formation of these microtissues by other less-spontaneous methods is necessary. For example, the use of spinner flasks is a well-established method for forming multicellular aggregates. Due to the constant mixing of the cell suspension by a stirrer, the cells are prevented from attaching to the wall of the flask and aggregate into microtissues [44]. Through variations in cell density or stirring speed, the size and number of microtissues can be tailored to specific needs. However, this method has the disadvantage that cell behavior and viability can be affected by constant shear stress [26, 45]. In turn, reduced cell viability or functionality could be detrimental for implantation of microtissues in vivo.

A possible solution to this problem is a method developed by NASA that involves the formation of spheroids using microgravity. This approach is based on a special rotary cell culture system that provides constant mixing of the cell suspension with minimal shear forces through the constant rotation of the outer wall of the bioreactor [46].

In addition to product quality, other requirements for microtissue generation for clinical applications are simplicity and reduced cost. The liquid overlay technique has the advantage that no special equipment is required because either nonadhesive lab ware [47] or normal lab ware coated with nonadhesive substances, such as agar [48] or agarose [49, 50], can be used. Given that cells do not adhere to the surface, they aggregate and form microtissues. However, this occurs in a rather uncontrolled way that can lead to high heterogeneity in the size of the microtissues [26]. Improved nonadhesion approaches ensure a more consistent and homogenous size distribution. The standardization of microtissue size, composition, and shape is of upmost importance in the clinical field. It can be achieved by seeding cells in nonadhesive microstructures generated by micromolding. Prominent inert materials that can be used to generate these microstructures include agarose and polyacrylamide [51].

The use of external forces, such as centrifugation, ultrasound, and magnetic or electrical forces, can induce or accelerate cell aggregation. By using low-speed centrifugation of a cell suspension in a conical tube, cells form an aggregate that may reach a relatively large diameter [52]. However, the main disadvantages of this approach are again shear forces and the difficulty of upscaling [21]. For the ultrasound-guided generation of multicellular aggregates, an ultrasound standing wave trap is utilized to direct cell aggregation [53]. The 3D aggregation of cells can also be achieved via positive dielectrophoresis [54]. Utilizing endocytosis of liposomes containing magnetic nanoparticles allows cell aggregation with magnetic forces [55]. However, while only minor adverse effects on cell physiology have been reported when applying external forces, these cannot be ruled out completely. Another shortcoming of such methods is the need for specialized equipment [26].

Microtissue production for regenerative therapy should be standardized, reproducible, and, most importantly, cost-effective to guarantee efficacy and, ultimately, translation into clinics. The described approaches, which use external forces (with the exception of low-speed centrifugation), including constant mixing of the cell suspension, have the disadvantage of needing special equipment, which increases production costs and lowers the common availability of these platforms. Because it uses common lab ware and reagents, the liquid overlay technique is a cost-effective and easily reproducible platform. However, the advanced and more standardized micromolding technique requires specialized facilities and therefore might not be easily transferrable to every lab [21, 26, 56].

The described methods have several shortcomings, such as variable microtissue size, difficulty of upscaling, and a harsh production environment with external forces. The production of microtissues using the hanging drop method can overcome some of these challenges because it is a very gentle method for the production of well-defined microtissues with a constant and reproducible cell number and size.

The hanging drop method is based on gravity-enforced cell assembly, which is commonly achieved by pipetting small drops of a cell suspension on the inner surface of a Petri dish lid. By turning the lid upside down, a hanging drop is formed and persists through surface tension. The cells in the drop accumulate, driven by gravity, at the liquid/air surface and form microtissues [57]. It has been shown that this procedure for forming microtissues is applicable for different cell sources including primary cells, such as cardiomyocytes [58]; stem and progenitor cells, such as embryonic or mesenchymal stem cells [16]; and immortalized tumor cell lines, such as MCF-7 [57]. The production of coculture microtissues with a defined composition is an advantage of the hanging drop method. Furthermore, several coculture and hybrid setups are feasible and comprise mixed microtissues; microtissues coated with another cell type; or so-called Janus microtissues, which are fused spheroids [40].

In summary, the hanging drop method using Petri dishes is a cost-effective, gentle method that guarantees reproducibility and does not require special equipment. However, it is difficult to upscale and is not standardized. To meet these requirements, more sophisticated hanging drop techniques exist.

### 3.2. Automation

To facilitate standardized production of microtissues, which is crucial for applications such as regenerative therapies or drug screening, an automated, high-throughput approach is indispensable.

While the upscaling of the hanging drop procedure in a Petri dish is inconvenient, several companies have recently developed systems that are suitable for high-throughput microtissue production [59, 60]. They have developed specific plates for the modified production of hanging drops that are based on multiwell plates with special wells that have holes at the bottom for the formation of a hanging drop. The great advantage of these novel systems is that they can be combined with liquid handling systems for the automated, standardized, high-throughput production of microtissues. These automated approaches also overcome the challenge of long-term cultivation, which is a main drawback of the conventional hanging drop culture. The accessibility of the hanging drop from the top enables media exchange, which facilitates long-term cultivation of microtissues in hanging drops [59]. These systems also simplify the addition of reagents to individual microtissues in a hanging drop, thereby substantially expanding the opportunity for potential experimental settings and treatments [59].

Although automated hanging drop microtissue production is rather expensive because of the need for special, patented lab ware, its ability for large-scale production and long-term cultivation of microtissues makes it a promising and important tool for a wide range of biomedical applications, such as cytotoxicity studies and, more importantly, tissue-engineering approaches for regenerative medicine.

### 3.3. Cryopreservation and Biobanking

For broad preclinical and clinical applicability in the setting of regenerative or other therapies, an off-the-shelf microtissue concept is desirable. One important feature of an off-the-shelf microtissue product is its instant availability for the aforementioned applications. Therefore, key aspects, such as storability and, in particular, cryopreservation, are of upmost importance [61].

Ice formation during cryopreservation may be a crucial and critical event because it can induce severe cell damage. While freezing is commonly associated with ice formation, ice can also be formed during thawing. Therefore, it is important to identify optimal settings for freezing and thawing [62]. In addition to the common problems with the cryopreservation of single cells, such as cryoinjury caused by ice formation [62], there are additional obstacles for the cryopreservation of 3D tissues. These include the microtissue size-related impaired diffusion of substances like cytokines or even gases, such as oxygen, to the inner part of microtissues [63]. Regarding this limited mass transfer to the microtissue core, the optimal incubation time of the cryoprotective agent (CPA) needs to be determined based on microtissue size and cell composition because a too-long incubation period may harm the cells through CPA cytotoxicity [64]. Importantly, the CPA should be distributed homogeneously within the entire microtissue to protect the cells from intracellular ice formation, which can lead to severe cell damage and cell death [64, 65]. On the other hand, a too-intense incubation with the CPA may lead to enhanced dehydration of the cells, which limits their capacity to recover after thawing [64].

During vitrification, liquids transform to a solid state without ice formation. This can be achieved by rapid cooling in combination with CPAs with a high osmolarity [64], resulting in the prevention of cryoinjury from ice formation [66]. However, the major drawbacks of this method are the use of CPAs in a high concentration, which can lead to osmotically induced cell damage [67], and the requirement for fast heat transfer to achieve rapid cooling rates, which makes it difficult to apply this method for larger-volume and high-throughput biobanking [68]. By contrast, the slow-cooling approach is accompanied by a reduced CPA cytotoxicity because lower concentrations are needed [64]. However, a critical step for slow cooling is determining the optimal cooling rate because suboptimal cooling rates may cause either slow- or rapid-cooling injury [69]. Moreover, every cell line has a specific optimal cooling rate [62]. The ideal cooling rate of tissues may also differ from their single-cell counterparts. It has been shown that gap junctions between cells propagate intercellular ice formation at a given temperature, which leads to increased intracellular ice formation and finally to cryoinjury [70]. With the help of special programmable freezers, it is possible to upscale the sample volume to freeze, which would be beneficial for cryobanking therapeutic microtissues. It has also been shown that it can be advantageous to control the ice nucleation by the addition of nucleants, such as cholesterol [71]. For some cell sources, such as neural or embryonic stem cells, freezing in aggregates results in higher numbers of recovering cells than what can be achieved with single-cell suspensions [72, 73]. This beneficial effect was attributed to the cryoprotective effect of the surrounding ECM [73]. In this context, it has been shown that 3D cell aggregates of neural or embryonic stem cells can be cryopreserved with a large quantity of cells recovering after thawing [72, 73].

Therefore, it seems promising that also other types of microtissues can be safely cryopreserved by individually adjusting all the parameters, such as CPA composition and concentration, cooling method and rate, thawing rates, and possible additives.

## 4. Microenvironment

While stem cell therapy is raising hope in the regenerative field, the use of single-cell suspensions is expected to fail at recapitulating the physiological 3D microenvironment of cells. It is essential to consider cells in their entire context because in nature, a cell is never without a surrounding that has profound influence on its properties [23, 74].

The unique microenvironment that is generated in microtissues is crucial for the benefits of this culturing method and can be considered to be the potential link to successful regenerative therapies. The following chapter aims to reveal the advantages of 3D spheroids relative to their microenvironment.

**Figure 1:**
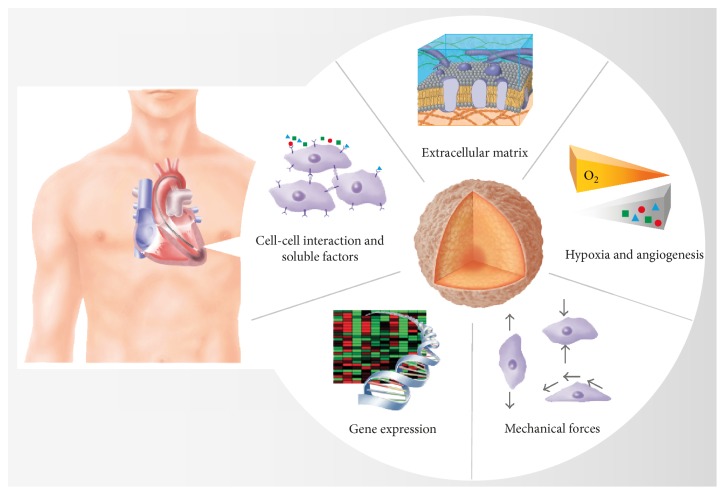
The concept of a scaffold-free, cell-based 3D microenvironment for advanced cardiac regeneration via transcatheter-guided, intramyocardial transplantation.

The biomimetic microenvironment that is sufficiently established in microtissues bears a strong resemblance to native tissues and is therefore capable of mimicking the real conditions found in an organism [21]. For this reason, microtissues are an excellent example of how cells themselves are the best tissue engineers [75]. The cultured cells benefit from the opportunity to interact with one another and to adhere to their own secreted ECM. The intricacy of extensive cues contributes to a more in vivo-like morphology, physiology, and behavior of the cells and therefore improves basic cell functions, including differentiation and proliferation [76–78]. Microtissues produce a microenvironment that displays the fundamental features of the environment produced in natural tissues and its critical impact on the resident cells (Figure 1).

### 4.1. Cell-Cell Interaction

Cell-cell interaction is a key requirement of tissue formation and repair and is almost completely missing in single-cell therapies. This could help to explain the lack of efficiency of such therapies, and for this reason, the current hypothesis is that enhanced cell-cell interaction, which is achieved in microtissues, may overcome this problem.

The general principle of spheroid generation is to establish an environment in which the intercellular forces are more powerful than the interaction of the cultured cells with the matrix molecules of the substrate [79]. Cell-cell contact seems to play a crucial role in the homeostasis of microtissues. Due to high cell density and the three-dimensional nature, communication with neighboring cells is possible at all sites of the cell membrane, and only low concentrations of signaling molecules are necessary to regulate the tightly packed cells [80]. Soluble molecules, such as growth factors, hormones, and cytokines, influence the cells in an autocrine or paracrine way. The crosstalk between cells may also occur directly via adhesion molecules, primarily cadherins [79]. Interestingly, some cell types, such as MSCs, do not normally express cadherins in conventional monolayer cultures but appear to do so during spheroid formation. It has been shown that without those adhesion molecules, cell aggregation is not possible, which makes them a key factor in the formation of microtissues [81].

By interacting with numerous receptors, adhesion molecules determine cell fate by controlling growth, proliferation, and survival. Cell death and apoptosis are governed by signals from neighboring cells. Importantly, omission of these survival stimulants is fatal [79, 82]. Another indication underlying the significance in the context of life and death is that the degradation of cellular contacts is one of the first signs indicating that a cell is undergoing apoptosis [79].

Intercellular networking enables the coordination of tissue formation and facilitates the adaptation to new circumstances as an integrated system. Such synchronized operations are omnipresent during embryogenesis and also during other naturally occurring processes, such as wound healing and tumor invasion, which require the cells to work en bloc [79]. This highlights even more the importance of these features in regeneration.

Particularly in cardiac tissue, cell-cell contacts have an unparalleled role that is well represented as intercalated discs between cardiomyocytes. Properly built cellular contacts ensure correct mechanical and electrical conduction, which results in synchronized contractions. In microtissues, cardiomyocytes can maintain this cell-specific capacity and exhibit even more differentiated electrical properties than when they are cultured as monolayers [58, 83].

Driven by intercellular communication, cells are competent to self-assemble in a certain organized order, which mimics their native, organotypic architecture [84]. The proper construction is generally predetermined by the cell-specific function.

Again, cardiospheres are an excellent illustration of this concept. It has been shown that their spontaneous formation is dependent on transforming growth factor *β*1 (TGF*β*1), which promotes epithelial-mesenchymal transition (EMT), a morphogenetic mechanism that plays a role not only in developmental biology but also in adult tissues to induce stem cell-like properties. Interestingly, EMT in cardiosphere formation suggests recapitulation of the mechanisms involved in heart morphogenesis and ischemic injury [85].

As another example, spheroids composed of endothelial cells (ECs) form an outer layer with differentiated cells with tight cellular contacts and an interior core with unorganized, apoptotic cells, with the final goal of building a lumen [82].

Cocultured spheroids of mixed cell populations can also show this self-organizing dynamic that leads to compartmentalization. In such aggregates, ECs, for example, always move to the periphery to form a boundary between the enclosed cells and the environment, similar to what they would do in the body [2, 21].

Interestingly, because all natural tissues contain several distinct cell types, mixed spheroids may reflect real biology even more closely. Heterotypic cooperation is essential for most cells to unlock their full potential [26]. Particularly in stem cell therapy, it is important to be aware of the different interactions and their consequences. For example, it is important to know that ECs transplanted in vivo are dependent on mural cells to build a stable vasculature system connected to the host [86, 87].

The multilevel influence of intercellular communication on distinctive cellular features highlights the importance of in vitro remodeling of this specific microenvironment. Fortunately, microtissues appear to implement this essential element efficiently.

### 4.2. Extracellular Matrix

Another significant feature that makes microtissues an effective technology to mimic natural tissues is the formation of ECM, into which the cells are incorporated. The cells themselves produce and remodel the components of their environment, reestablishing and resembling the conditions in vivo [25, 76]. Thanks to their enhanced ECM production, microtissues might therefore increase the chances of successful cell engraftment and tissue regeneration.

The fact that a pathologically modified ECM accompanies numerous diseases reflects the importance of ECM homeostasis in tissues. A typical example is the development of fibrosis after an MI, which aggravates disease progression [1]. Communication and crosstalk between the cells and the ECM in organs or microtissues are bidirectional and support signal mediation between cells. Normal cell physiology, including differentiation, proliferation, migration, and, most importantly, survival, is orchestrated not only by signals from their neighbors but also by interactions with the ECM [23, 26, 27, 76]. The ECM provides spatiotemporally coordinated cues in forms of chemical, mechanical, and hydrodynamic signals that have a strong impact on the cells [88]. Many cells are anchorage dependent, and anoikis describes the process in which apoptosis is provoked by the interruption of cell-matrix contact [22, 89].

Engagement of particular cell receptors with molecules of the ECM is translated and integrated into an intracellular signal, ultimately resulting in an altered gene expression pattern [27, 74]. Those signaling pathways are often the same as the ones activated by growth factors, indicating the involvement of the ECM components in the regulation of proliferative activity [90].

Furthermore, the ECM plays a key role in defining the shape of not only the tissue arrangement but also the cell morphology. Previous studies have shown that the cardiomyocyte phenotype depends on extracellular proteins, such as fibronectin or collagen. Disturbance of the well-balanced composition may result in hypertrophy [91, 92]. Along with the composition, the physical characteristics of different materials surrounding the cells, such as stiffness and elasticity, also determine their behavior [1].

In addition, the structural support of a rich ECM renders tissues more robust and resistant to mechanical stress. This is particularly important in regenerative medicine, where cells are required to withstand the handling procedures and transplantation to the target organ [93].

Subsequently, after implantation, the grafting of microtissues depends on adequate adhesion and functional integration into the host tissue. The ECM facilitates this process by providing the cells a temporary substrate to attach to and enabling the essential first step during cell transplantation [84, 94, 95].

Unlike monolayer cultures, harvesting of the microtissues does not require a harsh treatment with proteolytic enzymes. Therefore, the ECM can be conserved and pave the way for strengthened engraftment of the microtissues compared to their dissociated and disorganized counterparts [84, 89].

Taken together, these facts illustrate the importance of considering a cell in its overall context because there are no cells that are not surrounded by a matrix in natural tissue. Microtissues offer the unique opportunity to perceive the cell as a whole and to provide insight into all dimensions.

### 4.3. Angiogenesis and Hypoxia

As demonstrated, the biomimetic microenvironment represents a key feature for the overall strength of the microtissue approach. Another advantage arises from the specific design of those spheroids and relates to their angiogenic potential. Neoangiogenesis and revascularization after ischemic tissue damage is one of the main goals in regenerative medicine, and stem cells appear to be an important tool to induce neovascularization [75, 89, 96, 97]. However, vascularization remains a major issue in many tissue-engineering efforts to transfer such technologies into clinical routine. Microtissues may offer an attractive solution.

While every tissue with a thickness greater than 100 *μ*m, which exceeds the diffusion limitation, depends on its own vascular network for the supply of oxygen and nutrients to preserve cell viability, microtissues can be designed within a rationale size range to eliminate this problem completely [96, 98–101]. Furthermore, due to the limited mass transport within the microtissue, a hypoxic environment is created within the entire spheroid that is particularly present in the core.

Consequently, this leads to a preconditioning and adaption of the cells to the ischemic environment they will be exposed to after implantation. Their intent to survive forces them to adapt to the critical circumstances and might therefore shorten their adjustment time after transplantation. Several studies have revealed evidence for the improved resistance to hypoxia when cells were cultured as microtissues. The hypoxic conditions contribute to a predominance of pro- over antiapoptotic factors and therefore make the cells more resistant to cell death [15, 89, 102].

To become more resistant to hypoxic circumstances, various alterations at the gene expression level are available. Of particular relevance is the upregulation of the hypoxia-inducible factor-1-alpha (HIF-1*α*). It is the inductor of the expression of numerous growth factors, such as vascular endothelial growth factor (VEGF) and insulin-like growth factor-1 (IGF-1), which both have strong angiogenic potential [89, 99, 102, 103]. Different studies have verified a size-dependent secretion in microtissues that also indicates hypoxia as a powerful driving force of these factors [58, 99, 102]. The subsequent amplification of these substances profoundly stimulates the development of vessels in vitro and in vivo [17, 76, 89, 102–109].

However, the supply of these factors unaccompanied by any cells has been proven to be insufficient to establish a stable vascular network [101].

This makes microtissues the ideal candidates to deliver not only stem cells but also the necessary angiogenic factors to stimulate neovascularization and subsequent coupling to the host system [18, 103, 104, 110].

### 4.4. Niche-Like Conditions

The previous chapters have already demonstrated the multifaceted impact of the microenvironment on cellular physiology. An appropriate environment is often a precondition for cells to maintain their phenotype, including their differentiated gene expression levels. Every cell expresses only a certain repertoire of the whole genome that is specified for the distinct cell type. This individual spectrum of expressed genes can be further altered according to external circumstances, showing that the surrounding of a cell has a profound influence on its functionality [90]. In this regard, microtissues might reveal unprecedented conditions that are beneficial for the stem and progenitor cells used for clinical therapies.

It is widely acknowledged that cells can have dramatically changed expression patterns depending on whether they were cultured three-dimensionally or as monolayers [26, 74]. It has been proven that several cell types are prone to lose their most important characteristics when cultured in a two-dimensional manner. Examples underlying the fundamental impact of the environment on functionality include ECs losing their tight junctions and therefore their barrier function or hepatocytes, which become less viable and drop their key functions, such as detoxification [25, 26, 82, 111–114].

Interestingly, cardiac progenitors also show different gene expression profiles depending on their culture mode. Although progenitor populations are isolated with different isolation techniques and cultured with different medium conditions (c-Kit^+^, Sca-1^+^ cells, and CDCs), they display very similar gene expression patterns when cultured in monolayers. By contrast, 3D-cultured cardiospheres have been shown to upregulate the expression of genes encoded for signaling molecules, such as BMP-2, HGF, LIF, PTGS-2, VEGFA, and PDGFRB, which direct cardiac development, angiogenesis, and cardioprotection during heart failure [115].

In addition, in the setting of stem cells, the microenvironment seems to determine the functional capacity of the cells, in this case their stemness. Often called a stem cell “niche”, the surrounding of the cells is responsible for maintaining the majority of the cells in a quiescent state. It is the niche role to react to a perturbation, such as tissue injury. Active signal transmission to the progenitors might enable an organism to reestablish the destroyed balance by inducing proliferation and differentiation. Therefore, the stem cell niche, with its panoply of structural signals and different growth factors, has a profound impact on their plasticity, particularly the maintenance of multilineage differentiation, expansion, and migration [9, 22, 27, 28, 48, 50].

Stem cells can lose their pluripotency and self-renewal capacity when cultured as monolayers [94]. Several studies on MSCs have confirmed the maintenance of their expansion efficiency, the upregulation of pluripotency markers, and an enhanced differentiation capability along mesenchymal lineages, as well as their transdifferentiation into neuron- and hepatocyte-like cells when cultured as aggregates [81, 93, 94, 116]. One reason for this improved multipotency of MSCs might be epigenetic changes in pluripotency-associated genes induced by 3D cultivation [117].

In addition to cell-cell and cell-matrix contact, another contributor to the promotion of pluripotency present in microtissues is the aforementioned hypoxia [118, 119].

Another advantage of stem cells is their potential to migrate to injured tissues where they are needed. Stem cells in microtissues appear to have altered homing properties, leading to improved migration qualities [120, 121].

MSCs can also be stimulated to produce many different protective molecules, including anti-inflammatory factors. The given conditions in spheroid cultures seem to cause intracellular stress, leading to a MSC-induced self-activation of an anti-inflammatory response. Interestingly, higher amounts of these factors are already available in the microtissues before they are implanted into the inflammatory milieu [80, 122].

It is challenging to identify the individual cues and forces prevalent in this specialized niche, all of which contribute to the stemness of cells in a synchronized fashion. It would be an extremely arduous task to provide all these factors individually in an attempt to artificially mimic this niche [88, 113].

Therefore, the specific microenvironment of microtissues may better resemble the natural stem cell niche and may optimize the therapeutic efficiency of MSCs, which is closely related to conservation of their stemness [22, 81].

### 4.5. In Vitro Model for Cardiac Regeneration

To obtain a better insight into the basic biological processes, it is crucial to take into account all dimensions of nature that are barely present in the current two-dimensional approaches. Therefore, the biomimetic microenvironment of microtissues may represent a more relevant tool to study in vitro basic biology and physiology including cell interaction, adhesion, migration, and tissue formation [21, 25, 74]. These preliminary studies are necessary before implementation of a regenerative therapy in vivo, whether into animals or humans.

In embryology and developmental biology, three-dimensional spheroid models are already being used to identify the environmental cues that are crucial for morphogenesis. It has become evident that both preprogrammed gene expression patterns and extracellular impulses, such as mechanical forces, are responsible for correctly guiding the development [74].

Inspired by the use of microtissues in drug screening and tumor research, translational researchers are also interested in using these tools to study regeneration. For example, in cardiac regenerative medicine, the concept of microtissues may represent a suitable platform to examine cardiomyocytes in a controllable 3D milieu. It could efficiently provide insight into their pharmacological, electrophysiological, and intercellular behavior [123–128]. Microtissues with long-term contractility as well as the stable expression of cardiac-specific surface markers can be generated through the self-assembly of isolated cardiomyocytes [58, 124, 129]. The assembly of cells from heart biopsies produces a natural composition of heart tissue. These tissues have been used to determine principles of cellular interactions between cardiomyocytes and fibroblasts, which are significant in pathophysiology [129]. In addition, this spheroid assay could be used to find the necessary factors for directing the differentiation of stem cells into cardiomyocyte phenotypes [124]. Microtissues also represent an interesting in vitro model for graft-host interactions in cardiovascular regenerative medicine because engraftment and integration of transplanted cells are based on the same mechanisms behind aggregation [91].

## 5. Microtissues in Regenerative Medicine

### 5.1. Safety

A cell-based therapy has to be effective and safe. Some major concerns of stem cell-based microtissue treatments are severe immune reactions [108, 130], tumorigenicity [131, 132], embolism [42, 130, 132, 133], and the assumption that multicellular microtissues may cause microvascular obstruction that results in myocardial damage [130]. This chapter will focus on these safety concerns that are specifically attributed to microtissues in the preclinical and clinical setting of cardiac regeneration.

Autologous cell sources are of particular interest because immunologic rejection is avoided [130]. Nevertheless, there are functional, technical, and logistical drawbacks to using autologous cells, such as patient-specific variability, time-consuming cell expansion, and delayed availability. As an alternative, immediately applicable, well-characterized, and standardized allogeneic cell sources from healthy donors might be advantageous. However, the literature to date is contradictory regarding their in vivo immunogenicity. On the one hand, it has been shown that allogeneic immunoprivileged stem cells, such as MSCs, become immunogenic and change their secretory profile after differentiation [134, 135]. On the other hand, several clinical trials addressing cardiac regeneration have demonstrated that clinically relevant allogeneic cell types, such as MSCs, CDCs, and embryonic stem cell-derived cardiac progenitors, can be applied safely without provoking immunological rejection [13, 136, 137]. This might be explained by the fact that these cells are short lived and/or do not differentiate into immunogenic cells, such as cardiomyocytes. Moreover, they exert their regenerative potential primarily by paracrine effects. While the aforementioned studies address stem cell therapy with single-cell suspensions, the question of allogenicity might be different in the three-dimensional microtissue environment, which changes cell behavior and gene expression [21, 138]. Tseliou et al. addressed this question by characterizing the immunologic profile of allogeneic, cardiac-derived microtissues by evaluating their safety and efficacy in repairing ischemic heart tissue. Cardiospheres express a low immunogenic profile in vitro and inhibit the proliferation of alloreactive T cells. Syngeneic and allogeneic cardiospheres attenuate the inflammatory response that is histologically observed in the peri-infarct region. Furthermore, an increase in myocardial viability in an infarcted rat heart, as well as decreased scar size, improved cardiac function, and attenuated adverse remodeling, could be observed without deleterious immunological sequelae. The large functional and morphological benefits of allogeneic cardiospheres in acute MI highlight the therapeutic potential of these cardiac microtissues. Their hypoimmunogenic phenotype enables cardiospheres to evade alloimmune reactions and to modify the proinflammatory milieu created after MI [108]. While the question of immunogenicity has been addressed in stem cell therapy, it appears to be complex and controversial, and further investigation for both the 2D and 3D cell delivery formats is needed.

In addition to immunogenicity, tumorigenicity also represents a safety issue that has to be addressed. A 3D cell system using poly-L-ornithine seeded with putative cardiac stem cells isolated from the adult canine heart showed that transplanted cardiospheres survived transplantation conditions and did not form tumors even at 3 weeks posttransplantation. The cardiospheres displayed characteristics of undifferentiated cells and differentiating cardiomyocytes and/or vascular cells. Together with their ability to undergo differentiation into various cardiac cell types, as well as their relative resistance to oxidative stress, the lack of tumor induction in vivo supports cardiospheres as a suitable delivery system of stem cells for tissue regeneration [131].

Another concern that has been raised regarding the safety of microtissues is embolism. Preclinical studies in rodents and pigs indicate that the self-assembling microtissues may be more effective than dispersed CDCs. However, the more desirable intracoronary route has been assumed to be unsafe for cardiosphere delivery [130]. Cardiospheres are large (30–150 *μ*m), and given that capillaries have diameters of only 8 *μ*m, intracoronary delivery of cardiospheres was assumed to be implausible given the likelihood of coronary microembolization [42, 130, 132, 133]. A dose-ranging study in minipigs using optimized cardiosphere size was performed to assess the feasibility and safety of intracoronary cardiosphere infusion [130] as well as direct injection through thoracotomy [132]. There were no deaths (sudden or otherwise) in either group after the immediate periprocedural period. Moreover, postmortem examinations with gross analysis as well as histology of the heart, brain, kidney, lung, liver, and spleen detected no tumors 8 weeks after intramyocardial injection of CDCs or cardiospheres [132]. Therefore, intracoronary delivery of cardiospheres appears to be safe and also remarkably effective in decreasing scarring, arresting adverse remodeling, increasing myocardial perfusion, and improving hemodynamic status after MI [130].

While efficacy is highly desirable in stem cell therapy, it is first critical to thoroughly address safety issues. Microtissues have shown promising safety profiles thus far, which will hopefully be confirmed in future studies.

### 5.2. Spheroids as Stem Cell Vehicles

Regenerative medicine has been repeatedly suggested as a promising, next-generation approach to treat diseases because it tries to not only treat the symptoms but also address the root of the problem by replacing the lost cells and regenerating the diseased tissue. In this regard, stem cell-based therapies have raised substantial hope for curing degenerative diseases and damaged tissue. Preclinical studies have shown that such therapies can functionally improve diseased tissue after pathologic events, such as MI [139], osteoporotic bone loss [140], or cartilage lesions [141].

Microtissues, as an alternative cell format to single-cell therapies, have been launched as a powerful delivery tool to serve as stem cell vehicles. Furthermore, preclinical studies from numerous fields of regenerative medicine have demonstrated superior qualities to their single-cell analogs. Hepatocytes cultured as microtissues could avoid the loss of their specific gene expression and produce more detoxifying proteins when implanted into the liver. Chondrospheres could be successfully integrated into damaged cartilage without losing their differentiation capacity, which would occur if they were cultured as monolayers. Other examples are 3D aggregates of neurons, which are being tested as an alternative therapy for Parkinson's disease, or the transplantation of insulin-producing islets to treat diabetes mellitus [3, 141–145].

### 5.3. Cardiovascular Therapy

The following subchapter will discuss in more detail the potential of microtissues in the setting of cardiovascular regenerative medicine. Although it has been shown that the heart holds a certain capacity of self-renewal, it is evident that this regeneration potential cannot keep pace with the number of cells needed after cardiac injury, such as MI [6, 75, 146]. Therefore, not only a high cell number is essential to be delivered but also the cells need to attach to the host tissue, integrate, and survive to finally ensure long-term engraftment and efficacy [3].

In addition to the identification of the ideal cell source, it is crucial to optimize the delivery approach, including the route of delivery and, particularly, the cell format [3, 10]. Single-cell delivery has been the standard approach for cell-based therapies, and many different stem cell sources and delivery methods have been evaluated with promising outcomes in preclinical trials. However, despite successful advancement into the clinical setting, most of these approaches have failed to confirm the preclinical results and did not reveal substantial clinical evidence [3, 5, 10, 147]. One of the main obstacles for a successful translation into the clinical setting of cell-based therapies seems to be the poor engraftment and survival of the cells. The majority of the cells are washed out after transplantation, mainly because of venous drainage and contractions of the heart [17, 147–150].

To overcome these limitations, other cell delivery formats, such as self-assembled microtissues, have been proposed and investigated in several preclinical studies that have indicated several advantages when compared to their single-cell counterparts [121].

Microtissues provide the cells with an artificial network that behaves like the natural biology. The cells can reside within a microenvironment that contains vital cellular interactions and contact with ECM components. Therefore, they could serve as stem cell vehicles and ensure the attachment of the cells, which is the first prerequisite for long-term survival. Owing to their size, 3D cell aggregates are more likely to engraft within and subsequently adhere to the interstices of a tissue. This is attributed to their inherent ECM, which provides a temporary matrix to attach to [84, 107]. Various studies have demonstrated the greater (up to 14-fold) engraftment potential of different cell spheroids compared to single-cell suspensions when they are implanted intramuscularly or intramyocardially into rodents [17, 84, 94, 103, 107, 133]. Using microtissues as vehicles for the cells appears to be a promising alternative to guarantee an enhanced degree of mandatory engraftment, rendering the use of scaffolds, genetic modifications, or a supply of growth factors unnecessary [3].

After attaching to the host tissue, the next crucial step involves the appropriate integration and simultaneous inhibition of apoptosis despite the new, hostile microenvironment.

Unprotected single cells are predetermined to undergo apoptosis when entering the hypoxic and inflammatory milieu of an ischemic tissue [89, 149–151]. Several studies have attempted to prove the enhanced survivability of cells when cultured as microtissues and showed more abundant and fewer apoptotic transplanted cells in the host tissue [17, 107, 121, 152].

Another environmental factor that contributes to cell death after implantation is the inflammatory milieu [102]. Provoked by the enrichment of reactive oxygen species and other toxins in the ischemic context, the tissue is infiltrated by activated immune cells, which can even deteriorate the situation [153]. It has been proven that aggregation of stem cells could also enhance their anti-inflammatory characteristics, which could help them counteract this hostile environment [80].

A recent study of a preclinical porcine model showed that the intramyocardial injection of microtissues composed of MSCs caused increased retention and integration rates as well as improved survival compared to the corresponding single-cell suspensions [5, 10]. The study was carried out with a three-dimensional NOGA electromechanical mapping-guided approach [10]. The same transcatheter-based delivery of microtissues was also used in a model of ischemic cardiomyopathy, showing that microtissues could also be helpful in a chronic setting. This situation is even more difficult given that the acute homing signals are already absent and that the negative remodeling process has already taken place [133, 154]. In a study by Yee and colleagues, the delivered cells were not MSCs but cardiac progenitor cells (CPCs), which are an alternative cell source for cardiac repair. In addition to their ability to differentiate into vascular cells and contribute to angiogenesis through paracrine effects, they also hold the capacity to differentiate into cardiomyocytes and therefore contribute to direct regeneration of the heart [41, 42, 152, 155–157]. Cardiospheres are generated from biopsy material expanded in vitro and consist of different cell types, including progenitor cells surrounded by supporting cells and the ECM [152]. This composition mimics the natural niche in which the cardiac progenitors reside that is favorable for the survival and stemness of the cells [15, 121, 152, 158–160]. This particular niche seems to be essential for the stem cells to maintain their viability and differentiation potential [88]. Indeed, it has been demonstrated that spheroid culture led to an upregulation of stem cell-relevant factors, and the fraction of the c-Kit^+^ cells (which are thought to be the progenitors of the heart) increased over time [152, 160].

Another method for the formation of cardiospheres involves human pluripotent stem cells (hPSCs) and can avoid the need for biopsy. However, the usual techniques for enriching the fraction of cardiomyocytes from hPSCs are very laborious, dependent on antibodies or dyes, and still inefficient for generating the desired purity. This approach relies on the strong tendency of the cardiomyocytes to aggregate. Because the other cells in the hPSCs mixture do not share this property, the result is an enrichment of the surviving cardiomyocytes [161].

Several preclinical trials with cardiospheres in the setting of MI have revealed evidence for the efficacy of this cell type cultured as microtissues. Promotion of angiogenesis and attenuation of the inflammatory conditions as well as the increased engraftment resulted in improved cardiac function and the prevention of remodeling [108, 152].

Furthermore, it has been shown that the microtissue technique is applicable to different stem cell sources relevant to cardiac stem cell therapy [16].

Regenerative medicine of ischemic diseases aims at the initiation of neovascularization. It is evident that cardiac microtissues can only survive and function in the long run when their vascularization is ensured. Many cells in the damaged tissue are in a hibernating state due to ischemia and could be saved if early revascularization is supplied [97].

Stem cells, especially MSCs, reconstruct a vascular network through two mechanisms. On the one hand, they can differentiate into endothelial and vascular smooth muscle cells, which directly contribute to the formation of new vessel. On the other hand, they can facilitate angiogenesis through paracrine mechanisms, recruiting endogenous ECs to induce vascularization [109, 162–165].

Therefore, the efficacy of MSCs is mainly attributed to their paracrine effects. To address neovascularization during cell therapy, microtissues have immense potential because they can be modulated in composition and therefore in their functionality. They are able to secrete higher amounts of proangiogenic factors that they can also keep within the spheroids, preventing dilution within the growth media [58, 76, 89, 93, 102, 103, 108, 109, 121]. Microtissues made of rat cardiomyocytes have been shown to produce angiogenic factors in vitro [58] and are able to connect to the vascular system of chicken embryos after transplantation [18].

The beneficial effects on angiogenesis have been proven both in vitro and in vivo. Transplantation of different types of microtissues can lead to enhanced neovascularization and thus limb salvation in mice hind limb ischemia [89, 102]. Moreover, these cells have been proven to be beneficial in rodent MI models, where the induced vascular network supported the restoration of cardiac function and the attenuation of cardiac remodeling [17, 103, 107].

Another promising approach is the aggregation of mixed cell bodies. This method could foster further advances in the field of angiogenesis. By enveloping MSCs with a shell of ECs, microvessels can already be formed on the level of the 3D spheroid. Because ECs need contact with perivascular cells to become responsive to growth factors, they rely on a teammate. MSCs can fulfill this requirement by stabilizing the formed vessels and simultaneously providing the necessary growth factors. The efficiency of this innovative core-shell technique has also been observed in several in vivo models [76, 109].

Microtissues seem to have the potential to enrich and improve cell-based therapies. This has already been shown in preclinical small and large animal studies, as described above. Initial cell-based clinical trials for cardiac regeneration were performed with single cells and outlined the safety and feasibility, although they were not convincing regarding therapeutic efficiency [8]. One major reason for this is the poor engraftment of the injected cells, and therefore, next-generation cell therapies with improved 3D cell formats, such as microtissues, are needed [166]. However, to date, no clinical data investigating the impact of microtissues on tissue regeneration are available. However, given their diversity, simplicity, and reproducibility, microtissues as biomimetic vehicles appear to be a promising tool for further enhancing current stem cell therapy strategies.

### 5.4. Building Blocks: From Micro to Macro

Taking into account all the beneficial features of microtissues in stem cell therapy, it is clear that they might have the potential to be taken to the next level, which would be to use them to generate macrotissues. In tissue engineering, it is desirable to artificially design a perfect tissue or even duplicate an organ in vitro that could be successfully integrated into a host.

Given their capability to fuse with one another, 3D spheroids could exploit their full potential in larger tissue constructs by shifting the scale from micrometer to millimeter or even larger [93, 167]. In the complex field of tissue engineering, it is always desirable to mimic biological microenvironments. Natural tissues are generally built modularly by assembling organs from hierarchically organized units. The lobules of the liver, alveoli of the lungs, and nephrons of the kidneys are all excellent examples of this construction concept [25, 100].

Current methods of designing thicker tissues mainly involve scaffolds, which are very beneficial for tissues that need mechanical guidance to force them into a specific geometry, such as heart valves or blood vessels. More dynamic organs, such as the myocardium, might be impaired by the mostly rigid, shape-supporting materials [3, 58].

Scaffolds seeded with different cells bring the artificial tissue into the desired form. However, they are limited in the physiological integration of the multifaceted signals [167]. Because they are biomimetic and provide the appropriate setting to ensure phenotypes that are reminiscent of the cells in vivo, microtissues could serve as attractive building blocks and eliminate the need for scaffolds [77].

There is one major obstacle to engineering larger tissue implants that needs to be solved before a strategy can be successfully transferred to clinical practice. Cells within the artificial tissue construct must remain viable in vitro as well as in vivo after transplantation [1]. However, as diffusion is limited to a distance of 150–200 *μ*m, a blood vessel supply represents a prerequisite in tissues exceeding this thickness [96].

An implant can be equipped with purified angiogenic factors to promote the rapid recruitment of host cells to vascularize the tissue in situ, but controlling the release of these instable molecules remains challenging. In addition, it has been proven that chemical reagents alone are not sufficient to create a stable vasculature network [101, 167, 168].

Therefore, another strategy is to build the vascular network in vitro by colonizing the organoids with cells competent to establish inherent blood vessels. However, ECs are prone to undergo apoptosis when seeded into some matrices, such as collagen or matrigel [169]. In contrast, seeding ECs into the context of microtissues would be feasible because these cells survive in aggregates. By amplifying the angiogenic and differentiation potential of these cells, 3D spheroids could be the starting point for developing capillaries. Those could then be easily incorporated into the tissue and ensure that the required blood supply is available [96, 99]. Angiogenesis, like all processes in the body, takes place in all three dimensions and requires an environment that reflects this intricacy. In addition, microtissues also provide the mandatory ECM elements and a high cellular density to make spheroids the perfect vascularization units [25].

The efficiency of this strategy was demonstrated in a study where MSC spheroids were seeded on a scaffold, enabling the formation of a vasculature network connected to the host blood circulation system when implanted into dorsal skinfold chambers of mice [96].

When coated with ECs, microtissues could even improve vascularization. Thanks to the high degree of cellular self-organization in 3D, ECs already induce the formation of microvascular structures within the spheroids and are disposed to anastomose to the host network [25, 99].

More importantly, cardiomyocyte spheroids can be coated with either ECs or MSCs to create new blood vessels within the aggregates without the addition of any growth factor [97].

The feasibility of creating microhearts was demonstrated by Kelm et al., who were able to generate beating heart aggregates out of cardiomyocytes. In addition to the maintenance of a mature phenotype, the aggregates were also electrically coupled and showed the capacity to synchronize their beating frequency when various microtissues were planted together. Intermicrotissue forces that generate electrical continuity are essential for building larger tissues as well as for integration into the hosting heart without causing arrhythmias [58].

In addition, tissue-engineered blood vessels can be created with specific microtissues. This was shown in a fully autologous approach that was completely free of any foreign material. In an assembly device, the microtissue units, which were composed of fibroblasts and ECs, were manipulated to assemble into a tubular shape [170].

In addition to the need for an efficient vasculature, there are other issues in designing larger tissues in vitro that demand an alternative to scaffold-based approaches. Seeding scaffolds with cells rarely results in a sufficient and homogenous cell density, and the positioning of the cells remains difficult. However, the tissue construct needs to have a specific, organ-like shape, which is very challenging to accomplish without a scaffold [171].

3D spheroids may overcome these hurdles because their shape can be adapted according to their desired application, they naturally provide ECM, and they have a great potential for vascularization. All of these favorable characteristics qualify microtissues as appropriate tissue units [3].

However, it is necessary to precisely control the microtissue position in the complete system to obtain the optimal outcome. The use of bioprinting to achieve this control is raising significant hope in the field of tissue engineering. Microtissues are placed robotically using a tool similar to an inkjet printer. The positioning of the spheroids in all dimensions is controlled by a computer and results in complex tissue constructs or even whole organs composed of different cell types embedded in an ECM material [21, 93].

High precision, scalability, and the infinite combination possibility using different spheroids all suggest that microtissues could be the new pixels in tissue engineering [171].

## 6. Outlook

Regenerative medicine represents a growing field for a variety of diseases. Regenerative strategies are needed for cardiovascular disease in particular because the heart has only a limited capability of self-regeneration. Although numerous experimental and preclinical studies have demonstrated promising data, the outcomes of the initial clinical trials appear to be rather poor and indicate a lack of translation. Several key issues remain to be elucidated, including the ideal cell source and optimal cell format. In particular, the poor cell engraftment rate after transplantation of single-cell suspensions remains a major obstacle and needs to be addressed to enhance the therapeutic efficacy of cell-based concepts.

The unique tissue-like microenvironment that can be generated in microtissues may represent an interesting solution to increase cellular retention after delivery and subsequently enhance efficacy. In particular, the cellular environmental interactions mediated by neighboring cells and the secreted ECM may support physiological cell behavior and increase cell survival after transplantation because these environmental cues have crucial signaling properties. In addition, the hypoxic and inflammatory preconditioning within microtissues may further enhance homing, adaption, and, importantly, survival in the targeted injured tissue. These promising prerequisites of microtissues may pave the way for cell-based therapies with optimized entrapment, long-term retention, and improved clinical outcomes. In addition, the microenvironment within microtissues can be easily adjusted to the requirements of specific tissues by using and combining different cell sources. The resulting individual and unique microenvironments may have advantages for regenerative therapies for many diseases. Specifically, the combination of cell types with synergistic regenerative and paracrine effects might advance current strategies. The combination of MSCs and CPCs to treat chronic ischemic cardiomyopathy improves cardiac function more significantly than does treatment with MSCs alone, as shown in a recent preclinical trial [172]. These results provide evidence that next-generation cardiac cell therapies should include different cell types, and we envision that this effect could be enhanced when cells are delivered as microtissues, which would enable the close interplay between cell types. Depending on the cell source, the use of microtissues in regenerative medicine is suitable for both autologous personalized and allogeneic off-the-shelf therapies. Importantly, microtissues represent an ideal hybrid solution by combining the therapeutic advantages of both engineered tissues and single cells. On the one hand, they provide an advanced 3D cellular microenvironment, and on the other hand, they are still applicable via catheter-based approaches because of their size. As demonstrated by our group in a preclinical trial in the setting of MI, the spatially precise, catheter-based injection of engineered microtissues into ischemic cardiac target tissues is already feasible and safe [10]. After proving the feasibility and safety of this approach, the next step will be the investigation of its in vivo efficacy, including a head-to-head comparison with single cells. To translate this promising cell format from bench to bedside, it is important to perform proof-of-concept clinical studies. The newly established automated platforms for the standardized production and processing of microtissues may be an important tool to facilitate a GMP-compliant manufacturing process of the cell product. One of the remaining challenges to be addressed is the successful biobanking of microtissues of different cell types, which would reduce logistical issues, save money, and provide an immediately available off-the-shelf cell product. Next, to make cell therapy predictable and to study its fate in vivo, it is important to track the cell product after implantation. The visualization of injected single cells remains a challenging task that could be circumvented by the use of therapeutic microtissues, which consist of a large group of aggregated cells that can be easily labeled and detected. Our group has shown that microtissues composed of cells labeled with micron-sized paramagnetic iron oxide (MPIO) particles could be tracked in vivo using serial magnetic resonance imaging (MRI) after intramyocardial transplantation into a porcine heart [10]. We envision that the improvement of cardiac cell therapies could be achieved thanks to the tracking possibility of microtissues in vivo. This would allow their mechanism of action, their efficacy after injection, and their effect on cardiac regeneration to be studied.

In addition to the huge potential of microtissues for tissue regeneration, they may also serve as powerful building blocks for the generation of larger tissues or even whole organs, which is still an important need because of the general organ shortage in different medical fields. Microtissues also represent an interesting format to advance basic research. Their unique microenvironment, mimicking physiological cell behavior, can be utilized and modified to simulate and investigate diseases. While microtissues are an established model in tumor biology, they could play a significant role in the evaluation of various diseases at the interface between in vivo and in vitro. Finally, the combination of different microtissue-based microenvironments resembling different organs with a microfluidic system may provide the opportunity to study the physiological interplay between organs in complex multiorgan models in vitro [173]. Such a system could also be advantageous for drug testing in a setting that mimics the whole body [174].

## Figures and Tables

**Table 1:** Comparison of different 3D culture approaches.

3D culture systems	Technologies	Advantages	Limitations	Automated production	References
Scaffold-based	Biomaterials	Possibility of modification and activation (physical, chemical, and biological); adaptable scaffold size; mechanical support for damaged tissue	Foreign material (risk of foreign body reaction and overgrowth by fibroblasts and immunogenicity); biocompatibility of degradation products; degradation capacity; invasive implantation; special equipment	No	[1, 3, 5, 11, 17, 24, 26, 32–38, 56, 95, 96, 98, 111, 114]

Scaffold-free	Spinner flasks	Variation of cell density, stirring speed, size, and amount; large-scale production; long-term culture	Cell behavior and viability; constant shear stress; special equipment; high quantities of media	No	[2, 3, 21, 25, 26, 29, 44, 49, 56, 57, 79, 89, 116, 145]
				
	Microgravity	Constant mixing of cell suspension; minimal shear forces; large-scale production; long-term culture	Variation in size; special equipment (rotary cell culture system); high quantities of media	No	[2, 3, 21, 25, 26, 29, 46, 49, 56, 81, 161]
				
	Liquid overlay	No special equipment; several available methods	Coating (agarose, agar, poly-HEMA, and poly-D-lysine); variation in size and shape	No	[2, 3, 21, 25, 26, 29, 41, 47–50, 56, 57, 79, 85, 93, 97, 106, 108, 116, 130–133, 152, 156, 160, 161]
				
	Micromolding	Standardized size, composition, and shape; coculture possibility; defined composition; individual addition of reagents; monitoring and manipulation of single MTs	Media-exchange method; nutrient supply; special equipment (nonadhesive microstructure plates)	Yes	[2, 3, 15, 21, 25, 26, 29, 49, 51]
				
	Hanging drop	Standardized size, composition, and shape; no external forces; coculture possibility; defined composition; individual addition of reagents; monitoring and manipulation of single MTs	Media-exchange method; nutrient supply; special equipment (for automation)	Yes	[2, 3, 5, 10, 16, 18, 21, 25, 26, 29, 31, 40, 57–60, 80, 93, 96, 99, 102, 105, 110, 117, 120, 122, 123, 167]

## Abbreviations

CDC: Cardiosphere-derived cell
c-Kit: Tyrosine-protein kinase Kit
CPA: Cryoprotective agent
CPC: Cardiac progenitor cell
EC: Endothelial cell
ECM: Extracellular matrix
EPC: Endothelial progenitor cell
GMP: Good manufacturing practice
HIF-1*α*: Hypoxia-inducible factor-1-alpha
hPSC: Human pluripotent stem cell
IGF-1: Insulin-like growth factor-1
MI: Myocardial infarction
MPIO: Micron-sized paramagnetic iron oxide
MRI: Magnetic resonance imaging
MSC: Mesenchymal stem cell
VEGF: Vascular endothelial growth factor.
